# Use of the test-negative design to estimate the protective effect of a scalar immune measure: a simulation analysis

**DOI:** 10.1093/aje/kwag036

**Published:** 2026-03-04

**Authors:** Ziyuan Zhang, Christopher B Boyer, Marc Lipsitch

**Affiliations:** Department of Epidemiology, Center for Communicable Disease Dynamics, Harvard T.H. Chan School of Public Health, Boston, MA, United States; Department of Epidemiology, Center for Communicable Disease Dynamics, Harvard T.H. Chan School of Public Health, Boston, MA, United States; Department of Epidemiology, Center for Communicable Disease Dynamics, Harvard T.H. Chan School of Public Health, Boston, MA, United States; Department of Immunology and Infectious Diseases, Harvard T.H. Chan School of Public Health, Boston, MA, United States

**Keywords:** test-negative design, correlates of protection, generalized additive model, misspecification, waning

## Abstract

Exposure-proximal antibody levels, or scalar correlates of protection (COPs), are increasingly used to assess infection risk following vaccination or prior infection. A version of the test-negative design (TND), adapted from vaccine effectiveness studies, has been proposed to estimate this relationship, but its validity for continuous immune measures under realistic epidemic conditions remains unclear. We used individual-based transmission models incorporating waning and boosting immunity and simulated two scenarios: one with homogenous baseline risk and another with a high-risk group. Infection risk was modeled as a function of COP, both linearly and nonlinearly. Test-negative design samples were drawn from single or multiple days and analyzed using logistic regression and generalized additive models (GAMs). Model validity, defined as the ability to recover the true COP-infection incidence rate ratio relationship, was evaluated using mean absolute error. Transformed logistic regression recovered the true relationship when the correct functional form was known, including in the presence of confounding. When the parametric model was misspecified, GAMs outperformed logistic regression, particularly with large sample sizes and broad COP coverage. Because the true functional form is often unknown, flexible semiparametric approaches may be preferred in well-powered TND studies with antibody measurements.

## Introduction

Correlates of protection (COP) are measurable quantities, such as binding or neutralizing antibody concentrations that predict the degree of protection against incidence of an infectious disease. These markers provide valuable insights into the immune system's response to pathogens and vaccines,^1^ which is essential for advancing the understanding of immune mechanisms, as well as facilitating estimates of levels of protection in the population over time and informing the evaluation of new vaccines. For example, hemagglutination–inhibition antibody titers have been identified as a COP for influenza.^2^^,^^3^ Correlates of protection are particularly useful for estimating vaccine effectiveness by linking the magnitude of an immune response to levels of protection, especially in scenarios where direct measures of effectiveness are not available.^4^^,^^5^

Recent studies highlighted the importance of post-immunization antibody titers as effective COP for COVID-19 vaccines.^6^^,^^7^ Notable research efforts had investigated the use of these correlates to forecast absolute risks and relative risks of infection in randomized vaccine efficacy trials,^4^^,^^6^^,^^8-10^ using the antibody concentration measured at a fixed time post-vaccination. More recently, a prospective cohort study using a test-negative design (TND) analytic framework was employed to estimate the protective association of “exposure-proximal” COP, that is, how the COP level around the time an individual may be exposed to infection affects their risk of becoming infected.^11^^,^^12^

The use of the TND for exposure-proximal COP studies builds on a long tradition of using test-negative studies, which compare vaccination histories of those who test positive for a condition (eg, COVID-19) with those experiencing the same symptoms but testing negative for the condition, to evaluate vaccine effectiveness. If the vaccine provides all-or-nothing protection and there is no unmeasured confounding related to infection or test-seeking behavior, then the TND can yield valid estimates. Under these conditions, the odds ratio (OR) for vaccination among test-positive versus test-negative participants is an unbiased estimator of the incidence rate ratio (IRR), and one minus the OR provides the corresponding estimate of vaccine effectiveness. Several important assumptions are made,^13^ and methodological work has highlighted that they may be violated in practice.^14-20^

This area has recently begun to receive attention, including foundational work by Dunning introducing the scaled logit framework for modeling continuous protection functions, concurrent work by Middleton and Larremore extending this approach to TND data, and by related work from Follmann and Dodd introducing an imputation-based framework for pathogen-specific vaccine-induced immune responses that may have already been boosted by infection when individuals are identified for study inclusion.^21-23^ Our study provides an independent and complementary simulation-based assessment of the TND's validity under realistic epidemic conditions and under the assumption that such boosting has not yet occurred and that immune responses may or may not be vaccine-induced (like Middleton and Larremore, in contrast to Follmann and Dodd). We defined validity as the ability of a statistical model fitted to TND-sampled data to recover the true relationship between COP levels and infection risk. To reflect the constraints typically encountered in real-world TND studies, we evaluated validity from multiple perspectives, including functional form specification, sample size, and timing of data collection. The study also highlights how selection bias intrinsic to the TND sampling scheme and the bias from depletion of susceptibles in the context of leaky protection can influence the accuracy of TND-based inference.^24^

To operationalize these sources of bias, we designed simulations in which selection bias is inherently represented through TND sampling of symptomatically tested individuals, and depletion of susceptibles arises naturally through repeated infection and time-varying immunity within the transmission model. Two novel simulation scenarios were introduced to mirror disease transmission within a community: the first scenario incorporated vaccination and waning immunity from prior infections, and the second scenario built on this by additionally including population groups with varying levels of infection risk at baseline. These scenarios were designed to evaluate the performance of TND-based models in estimating IRR–COP relationship and to identify which statistical models and transformations most accurately captured it.

## Methods

### Model setup

**Figure 1:**
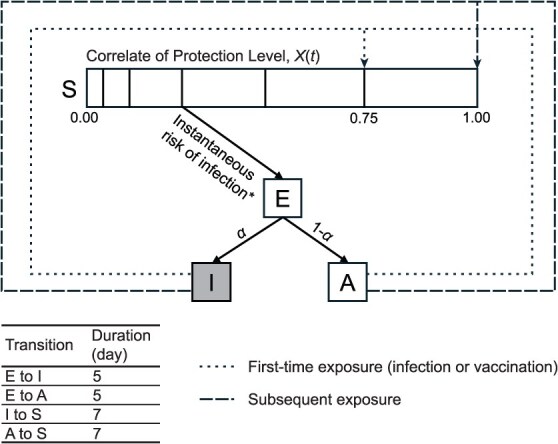
Schematic representation of the model. For detailed descriptions of the parameters used, refer to Table 1. * Instantaneous risk of infection: scenario 1, $\gamma \times \left(1-\psi \left(X(t)\right)\right)\times \left(I(t)+A(t)\right)$; scenario 2, $\gamma \times \left(1-\psi \left(X(t)\right)\right)\times \left(I(t)+A(t)\right)\times R$. State: S, Susceptible; E, Exposed; I, Symptomatically Infectious; A, Asymptomatically Infectious. Shade: Gray compartment, not eligible for vaccination; white compartments, eligible for vaccination.

We constructed an individual-based transmission model, involving susceptible, exposed, asymptomatically infectious, symptomatically infectious, and vaccinated individuals, to simulate disease transmission over 600 days within a community of 50 000 individuals (Figure 1), with detailed parameters listed in Table 1. The individual-based model allowed us to track the within-host dynamics of a scalar measure of immunity, denoted as $X$, that varied over time and affected an individual's risk of becoming infected. This dynamic framework naturally led to gradual depletion of susceptibles over time, allowing assessment of whether this process influenced estimates derived from the TND.

**Table 1:** Model parameters.

**Parameter**	**Description**	**Value**
*N*	Population size	50 000
*t*_latent_	Latent period	5 days^39^
*t*_infectious_	Infectious period	7 days^40^
*t*_revaccination_	Minimum interval between vaccine doses	56 days (8 weeks)^41^
*α*	Symptomatically infectious proportion	$1/3$ ^25^
*R*_0_	Basic reproduction number	2^42^
*γ*	Transmission coefficient	${R}_0/N(t)\bullet{t}_{\mathrm{infectious}}$
*λ*	Per capita rate of infection	γ * no. of infectious people
*w*	Antibody level waning rate	0.01 units/day
p_high-risk_	Proportion of high-risk individuals	Scenario 1, 0.0; Scenario 2, 0.2
Initial S	No. of initial susceptible cases	Without prior exposure: 48 950; With prior exposure: 1000
Initial E	No. of initial exposed cases	25
Initial A	No. of initial asymptomatic infectious cases	$\mathrm{Binomial}\left(25,2/3\right)$
Initial I	No. of initial symptomatic infectious cases	$\mathrm{Binomial}\left(25,1/3\right)$

In both scenarios, one-third of the infectious individuals were assumed to be symptomatic and this proportion did not vary over time or across subgroups.^25^ We assumed that the COP provided equal protection against asymptomatic and symptomatic infections. Individuals were not eligible for vaccinations while being symptomatically infectious and became eligible for revaccination if at least 56 days (8 weeks) had passed since their most recent vaccine dose, and they were not currently symptomatic.

All susceptible individuals without prior exposure started with a COP level at 0.00 units. A first-time exposure, including recovery after infection or vaccination, would raise the COP level from 0.00 to 0.75 units. With subsequent exposures, this level would boost from its current level to 1.00 unit. These specific values act as simplified indicators to assess the degree of protection conferred against infections following the first^26^^,^^27^ and subsequent^28-30^ exposures, respectively.

Lastly, all uninfected individuals experienced a linear immunity decline at a rate of 0.01 units per day, modeled as a simplified waning mechanism, whereas exposed and infectious individuals maintained their antibody level unchanged until it increased as described above upon recovery.^31-34^ Accordingly, antibody levels measured at the time of sampling were assumed to represent individuals' exposure-proximal immune state, reflecting their protection level immediately prior to infection. Data on each individual's antibody level and infection status were recorded on predetermined simulation dates. In the simulation, 0.5% of the eligible population was vaccinated every 15 days from day 1 to day 600, totaling 40 rounds. By the end of the simulation, 20% of the eligible population had received at least one vaccine dose.

### COP protection functions

Under scenario 1, the instantaneous risk of infection is given by


(1) \begin{equation*} \gamma \times \left(1-\psi \left(X(t)\right)\right)\times \left(I(t)+A(t)\right) \end{equation*}

where $\gamma$ is the transmission coefficient; $X(t)$ is the rescaled, time-varying COP level, ranging from 0.00 to 1.00; and $I(t)$ and $A(t)$ denote the numbers of symptomatically and asymptomatically infectious individuals, respectively. The function $\psi \left(X(t)\right)$ is the time-invariant protection function capturing the relationship between instantaneous risk of infection and COP level. All protection functions were constrained such that $\psi (0)=0$ and $\psi \left(X(t)\right)\ge 0$, with values increasing monotonically from 0.00 to 1.00 as $X(t)$ increases. As our primary specification, we assumed that risk decreased linearly with the COP level, that is $\psi \left(X(t)\right)=X(t)$.

In this discrete-time model, infection is represented as a per-time-step probability conditional on susceptibility, equivalent to a discrete-time hazard. Thus, the IRR approximates the instantaneous risk ratio in the continuous-time limit. Under this formulation, the IRR comparing $ X(t) $ to $ {X}^{\prime }(t) $


(2) \begin{align*} \notag\mathrm{IRR}\left(X(t),{X}^{\prime }(t)\right)&=\frac{\gamma \times \left(1-\psi \left(X(t)\right)\right)\times \left(I(t)+A(t)\right)}{\gamma \times \left(1-\psi \left({X}^{\prime }(t)\right)\right)\times \left(I(t)+A(t)\right)}\\& =\frac{\left(1-\psi \left(X(t)\right)\right)}{\left(1-\psi \left({X}^{\prime }(t)\right)\right)}=\frac{1-X(t)}{1-{X}^{\prime }(t)}\ \end{align*}

Taking ${X}^{\prime }(t)=0$ gives $\mathrm{IRR}\left(X(t),0\right)=1-X(t)$.

Additionally, we considered two alternative protection functions that applied nonlinear transformations to the COP level: a squared function, $\psi \left(X(t)\right)=X{(t)}^2$, and a cubic function, $\psi \left(X(t)\right)=X{(t)}^3$, for later sensitivity analyses. These formulations allowed us to assess the robustness of TND estimation approaches in recovering the shape of the relationship between COP and incidence when the relationship was more complex and nonlinear.^35^^,^^36^

### Confounding by risk category

In scenario 2, we modified our model to delineate between individuals in two intrinsic risk categories, representing differences in baseline exposure propensity (eg, behavioral or occupational factors) under the same transmission coefficient, $\gamma$. In the first category, the instantaneous risk of infection followed the same formulation as in Equation (1). In the second category, the instantaneous risk of infection was twice as high at a given COP level. This implies that for a given individual the risk may be written as $\gamma \times \left(1-\psi \left(X(t)\right)\right)\times \left(I(t)+A(t)\right)\times R$ where $R$ is the numeric representation of risk level, with


$$ R=\left\{\begin{array}{@{}c}1,\mathrm{if}\ \mathrm{low}-\mathrm{risk}\\{}2,\mathrm{if}\ \mathrm{high}-\mathrm{risk}.\end{array}\right. $$

This scenario was designed to assess the potential for TND estimation approaches to recover the relationship between COP and infection risk under a dynamic time-dependent confounding structure. Initially, one-fifth of the population was assumed to be high-risk, and individuals’ antibody levels was independent of risk category. However, as the simulation progressed, individuals in the high-risk category were infected more frequently, leading to faster accumulation of immunity among these individuals. Consequently, risk category became associated with higher COP levels over time, confounding the true relationship between immunity and infection risk (Figure S1). Risk group was known and fixed for each individual but was not used in matching; case–control pairs were matched only on sampling day.

### TND sampling scheme

In our simulation, we implemented a data-sampling scheme modeled after a canonical TND study, in which each symptomatically infectious case was matched with four symptomatic controls who were susceptible or exposed.^24^ By restricting sampling to symptomatically tested individuals, this setup incorporated the selection mechanism inherent to the TND, reflecting the conditioning on healthcare-seeking behavior that defines the design. This sampling scheme implies selection of controls is independent of COP level on that day. COP levels were assessed at the time of sampling, reflecting the exposure-proximal COP. It was assumed that all symptomatically infectious individuals tested positive, and that the matched controls were symptomatic but tested negative. TND datasets were drawn from specific sampling dates within this simulation, including single-day and aggregated-day periods, as detailed in the section “Statistical analysis.” Under this sampling scheme, the OR provides an unbiased estimate of the IRR of infection comparing vaccinated versus unvaccinated individuals.^13^ To increase sample size by aggregating data across multiple days, we used an incidence density sampling approach, selecting controls from individuals who remained at risk on the same day each case occurred.^37^

### Statistical analysis

Our analysis considered four regression models for each simulation scenario: two logistic regression models (with and without transformation of the independent variable, the COP measurement $X$) and two generalized additive models (GAMs, with and without transformation of $X$). In each model, infection outcomes were treated as Bernoulli-distributed variables, and parameter estimation was performed under the corresponding binomial likelihood framework inherent to logistic regression and GAMs.

For scenario 1, the untransformed models were specified as $\mathrm{logit}(p)={\beta}_0+{\beta}_1X$ for logistic regression and $\mathrm{logit}(p)={\beta}_0+f(X)$ for the GAM, where $p$ denotes the probability of infection.


(3) \begin{equation*} f(X)=\sum_{m=1}^K{\beta}_m{b}_m(X) \end{equation*}

is a smooth function based on thin plate spline basis expansions, where $K$ is the basis dimension and $m$ indexes the spline basis functions, ${b}_m(X)$ are the spline basis functions, and $\beta =\left({\beta}_1,\dots, {\beta}_K\right)$. In our implementation, GAM smooth terms were constructed with a basis dimension of 5. The transformed models applied a natural log transformation to capture the theoretical infection risk pattern and were specified as $\mathrm{logit}(p)={\beta}_0+{\beta}_1\ln \left(1-X\right)$ for the transformed logistic regression and $\mathrm{logit}(p)={\beta}_0+f\left(\ln \left(1-X\right)\right)$ for the transformed GAM. The rationale for the transformation was as follows: Under TND sampling, the IRR comparing two levels of COP satisfies $\mathrm{IRR}\left(X(t),{X}^{\prime }(t)\right)=\mathrm{OR}\left(X(t),{X}^{\prime }(t)\right)$. As shown previously, when the baseline COP level is defined as $X(t)=0$, we have $\mathrm{IRR}\left(X(t),0\right)=1-X(t)$for a simple linear protection function. The log transformation then allows for simple mapping between regression coefficients (which are on log odds scale) and protection function. This same rationale extends to the GAM framework, where the log odds of infection is modeled as a smooth function of $\ln \left(1-X(t)\right)$. In sensitivity analyses, we consider squared and cubic protection functions and use similar transformed and untransformed specifications. Using squared protection functions as an example, three scenarios were applied: (1) $\mathrm{logit}(p)={\beta}_0+{\beta}_1X$ and $\mathrm{logit}(p)={\beta}_0+f(X)$ for untransformed, misspecified logistic regression and untransformed GAM; Eq. (2) $\mathrm{logit}(p)={\beta}_0+{\beta}_1\ln \left(1-X\right)$ and $\mathrm{logit}(p)={\beta}_0+f\left(\ln \left(1-X\right)\right)$for transformed, misspecified logistic regression and partially transformed GAM; and Eq. (3) $\mathrm{logit}(p)={\beta}_0+{\beta}_1\ln \left(1-{X}^2\right)$ and $\mathrm{logit}(p)={\beta}_0+f\left(\ln \left(1-{X}^2\right)\right)$ for transformed, correctly specified logistic regression and fully transformed GAM.

Using a similar rationale for scenario 2, which incorporated heterogeneous risk levels, we have $\mathrm{odds}\left(X(t)\right)=\mathrm{OR}\left(X(t),0\right)=\mathrm{IRR}\left(X(t),0\right)=\left(1-X(t)\right)\times R$, where the baseline group for OR and IRR is the low-risk group with a COP of 0. Risk group was treated as a known baseline characteristic and was incorporated into the regression models as a main effect with COP to adjust for differential baseline risk. The untransformed models were specified as $\mathrm{logit}(p)={\beta}_0+{\beta}_1X+{\beta}_2I\left(R=2\right)$ for logistic regression, which adjusts for differential baseline risk by risk group, and $\mathrm{logit}(p)={\beta}_0+f(X)+{\beta}_2I\left(R=2\right)$for the GAM. This setup includes a smooth function of COP with a main effect of risk groups, mimicking scenarios where the exact functional form of the relationship is unknown. The transformed models were specified as $\mathrm{logit}(p)={\beta}_0+{\beta}_1\ln \left(1-X\right)+{\beta}_2I\left(R=2\right)$ for the transformed logistic regression and $\mathrm{logit}(p)={\beta}_0+f\left(\ln \left(1-X\right)\right)+{\beta}_2I\left(R=2\right)$ for the transformed GAM.

To assess the performance of all statistical models used for scenario 1 and scenario 2 under different sampling conditions, we conducted 1000 simulation repetitions, a number chosen to ensure stable estimates based on convergence of the mean absolute error (MAE). We examined the impact of sampling time points by training models on data from two pandemic phases: Early (days 50-140) and late (days 500-590), both collected at 10-day intervals. Additionally, we included single-day data (day 150 and day 600) to compare performance between continuous and isolated sampling. To evaluate the effect of sample size, we trained models with varying case numbers. For aggregated-day data, we sampled 300, 1250, and 5000 cases, while for single-day data, we sampled 125 and 500 cases, mimicking scenarios of sufficient and insufficient data availability. Model accuracy, and thereby validity, was assessed and compared using the MAE, computed in two steps. First, within each simulation, we calculated the mean absolute difference between predicted values and the true IRR across all immunity levels. Then, we averaged these values across the 1000 simulations to obtain a final performance estimate for each model under each condition.

## Results

### Scenario 1

**Figure 2:**
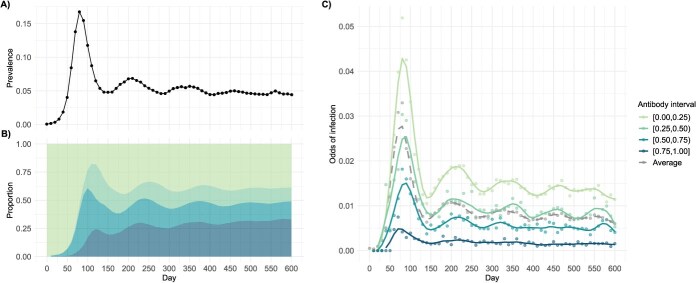
Trends in infectious prevalence, COP proportions, and odds of infection from simulation days 10 to 600 with an increment of 10. The odds of infection curves are fitted using cubic splines with 15 degrees of freedom. Panel A: the prevalence of infectious individuals. Panel B: the distribution of the population across different antibody intervals. Panel C: the odds of incident infection by COP intervals. Abbreviation: COP, correlates of protection.

During the 600-day period, five waves of infection were observed, with wave amplitudes gradually diminishing over time, displaying a transition toward a stabilized endemic state (Figure 2). Declines in the number of individuals with high antibody levels, primarily due to waning immunity, consistently preceded the emergence of subsequent waves (Figure 2A and B). As expected, individuals with higher levels of COP experienced lower mean infection odds, reduced temporal variability, and smaller wave amplitudes (Figure 2C).

**Figure 3:**
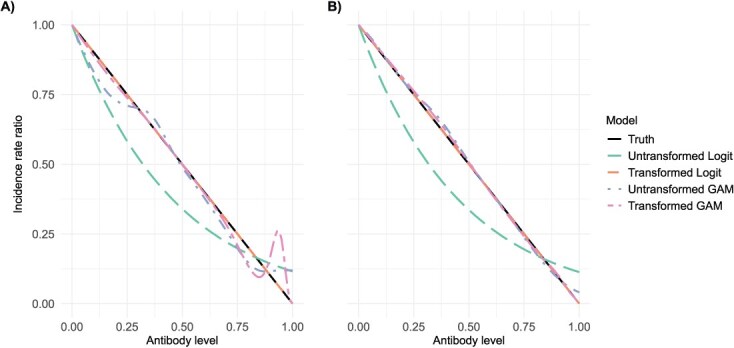
Performance of exposure-proximal TND regression estimators using data from a single simulated collection day versus aggregated collection days Panel A used data collected from simulation day 600 and panel B used data from simulation days 500 to 590 with an increment of 10. Abbreviation: TND, test-negative design.

**Figure 4:**
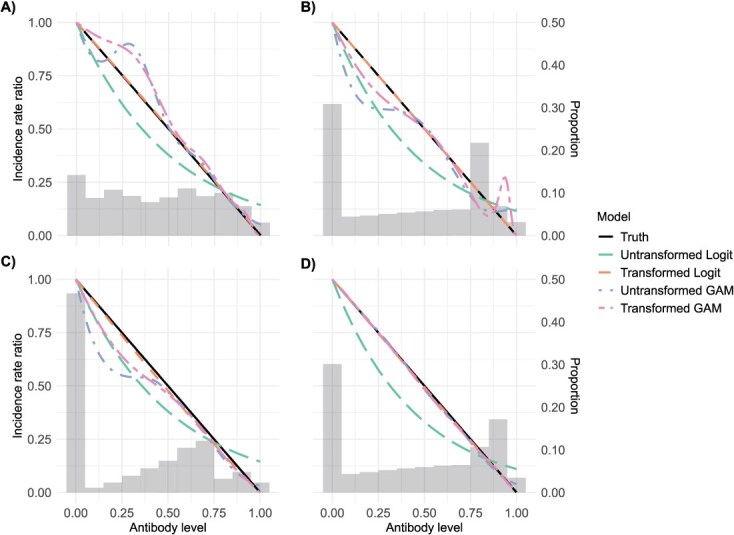
Impact of antibody distribution (gray areas in the plots indicate the proportions of COP levels within the population) and data aggregation on the performance of exposure-proximal TND regression estimators. Panel A used data collected from simulation day 150, panel B used data collected from simulation day 600, panel C used data from simulation days 50-140 with an increment of 10, and panel D used data from simulation days 500-590 with an increment of 10. Abbreviations: COP, correlates of protection; TND, test-negative design.

Figure 2 presents data from a single representative simulation run, chosen for illustration purposes. All subsequent figures (Figures 3 and 4 and Supplementary Figures S2 and S3) also reflect results from a single simulation to visualize key patterns, while summary statistics MAEs reported are derived from 1000 simulation repetitions.

Test-negative design sampling from the simulation showed that, when using data collected from a single day, the transformed logistic model accurately recovered the linear relationship between COP level and IRR. In contrast, the untransformed logistic model failed to do so due to misspecification of the functional form (Figure 3). Although both semiparametric GAM models generally captured the relationship, they were noisier, particularly at the highest COP values where the risk of infection is small and therefore subject to sparse events (Figure 3A). When applied to incidence density data aggregated across multiple time points, both GAM models generated predictions that more closely aligned with those of the transformed logistic model (Figure 3B), in contrast to models fitted using data from a single simulation day.

Besides the transformed logistic regression, both GAMs accurately estimate the IRR when large sample size are aggregated from multiple days using incidence-density sampling (Figure 4D). This accuracy is achieved during periods when the pandemic has stabilized after an extended onset, resulting in a diverse and adequately distributed range of COP levels. In situations with lower numbers of participants with certain COP levels (Figure 4A and C) or small sample size overall (Figure 4A and B) from TND sampling, GAMs tend to produce more complex estimated relationships, likely due to overfitting the noise in the data. In contrast, the transformed logistic regression consistently recovers the linear relationship.

The sensitivity analyses examined the ability of models to predict the nonlinear relationships between infection risk and COP levels, including squared and cubic functions, with sufficient data sampled. The curves generated from the correctly specified transformed logistic regression models accurately captured the true relationships, as in the previous analysis. Demonstrating their robustness, all GAM specifications (with and without transformations) also captured the relationship between infection risk and COP levels, with slight fluctuations around the actual lines (Figure S2).

Generally, within each pandemic phase and data source (aggregated- or single-day), models trained with larger sample sizes exhibited lower average MAEs than those trained with smaller sample sizes (Table 2). Similarly, models trained on aggregated-day data tend to yield smaller average MAEs compared to those trained on single-day data. Across most models, average MAEs are lower in the late pandemic phase (days 500-590 and day 600) than in the early phase (days 50-140 and day 150), except for the untransformed logit model. The smallest MAE (0.0057) was observed in the correctly transformed logit model trained on the largest sample size, 5000 cases, from aggregated-day data during the late pandemic phase. In contrast, the largest MAE (0.1617) was produced by the untransformed GAM model trained on 125 cases from single-day data on day 150.

**Table 2:** Averaged mean absolute error^a^ of models trained on aggregated or single-day data across early and late pandemic phases with varying sample sizes.

**Sample day**	**Early aggregated days (days 50-140)**			**Early single day (day 150)**		**Late aggregated days (days 500-590)**			**Late single day (days 600)**	
**Number of cases**	**300**	**1250**	**5000**	**125**	**500**	**300**	**1250**	**5000**	**125**	**500**
Untransformed logit	0.0920	0.0900	0.0899	0.0833	0.0758	0.1130	0.1126	0.1119	0.1191	0.1177
Transformed logit	0.0256	0.0177	0.0169	0.0460	0.0327	0.0209	0.0104	0.0057	0.0373	0.0214
Untransformed GAM	0.0769	0.0738	0.0758	0.1617	0.1695	0.0587	0.0345	0.0210	0.0968	0.0586
Transformed GAM	0.0325	0.0258	0.0328	0.0758	0.0710	0.0270	0.0149	0.0081	0.0843	0.0785

Abbreviation: GAM, generalized additive model.

a 1000 simulations for each model.

Among the four models, the transformed logit model consistently yields the smallest average MAEs, followed by the transformed GAM. In contrast, the untransformed GAM and untransformed logit models trained on early pandemic phase data and single-day data from the late pandemic phase exhibit relatively larger average MAEs. Notably, the performance of the untransformed GAM model improves when trained on late-pandemic aggregated-day data, where its average MAEs are comparable to those of the transformed GAM (Table 2).

### Scenario 2

Similar to scenario 1, scenario 2 showed that after stratifying by group of risk level, the transformed logistic model accurately predicted the linear relationship between COP level and IRR for each of the risk level group. Both semiparametric GAM models approximated the relationship, while sometimes deviating from the true pattern, and accuracy improved by fitting to aggregated data. The functional form misspecification in untransformed logistic model made it consistently produce the wrong shape and failed to capture the relationship (Figure S3).

Regarding MAEs, results from scenario 2 were consistent with those from scenario 1. Models trained on larger sample sizes and aggregated-day data consistently yielded lower average MAEs compared to those trained on smaller samples or single-day data. Performance generally improved in the late pandemic phase, with the exception of the untransformed logistic model. The transformed logistic model achieved the lowest MAE (0.0060) when trained on 5000 cases from aggregated late-phase data in the low-risk group, whereas the highest MAE (0.3385) was observed for the untransformed GAM model trained on 125 cases from single-day early-phase data in the high-risk group. Overall, the transformed logistic model performed best, followed by the transformed GAM. The untransformed logistic and GAM models showed higher MAEs, though the untransformed GAM's performance improved with late-phase aggregated data, becoming comparable to that of the transformed GAM. Additionally, while all models were trained on sampled data containing both risk groups, the MAEs were generally higher when predictions were evaluated on high-risk individuals compared to low-risk individuals (Table S1).

## Discussion

Antibody levels have been used in prior studies to predict the infection risk of influenza and COVID-19 and are considered important COPs for investigating the relationship between infection and individual immunity. However, apart from one recent paper designed for cohort studies,^11^ there has been little work to define approaches for estimating exposure-proximal COP, that is how an individual's instantaneously measured level of immunity predicts their susceptibility to infection at that moment. While prospective cohorts offer methodological strengths, case-control designs such as the TND are widely employed due to their comparatively low cost and feasibility. Thus, we evaluated when TND-based regression models can validly recover the true COP–IRR relationship under realistic epidemic dynamics. In our analysis, validity was quantitatively compared using MAE to evaluate how well each modeling approach recovered the true relationship. In related work, Middleton and Larremore proposed a scaled logit model that modifies the outcome function of logistic regression to flexibly capture sigmoidal protection curves and imperfect maximum protection.^21^ In contrast, our study focuses on transforming the exposure variable and applying semiparametric GAMs to recover linear and nonlinear protection functions and to accommodate unknown functional forms. While the scaled logit model has an intuitively appealing sigmoidal form, it remains a relatively restrictive parametric specification, extending the link function to improve fit in static datasets. Our approach evaluates transformations of the exposure variable within dynamic transmission simulations that include waning, boosting, and heterogeneous baseline risk. These complementary approaches therefore address different sources of model misspecification and offer alternative strategies for improving inference on COP in TNDs. An important direction for future work is to explore hybrid estimators that combine biologically motivated parametric structure, such as scaled link functions, with the functional flexibility of semiparametric approaches. Other related frameworks, such as the imputation-based approach by Follmann and Dodd, address complementary questions when immune responses are unobserved.

In scenario 1, correctly specified logistic regression produced estimates that were indistinguishable from the simulation's ground truth. GAMs likewise yielded valid estimates but were noisier and required larger samples and aggregated data. On the other hand, misspecified logistic models could not capture the shape of the relationship. In scenario 2, which introduced heterogeneous baseline risk, the performance of all statistical models replicates the general pattern of scenario 1 within both groups. These findings affirm that our results from scenario 1 extend to settings with heterogeneous risk subgroups, with stratification or subgroup indicators preserving the benefits of parametric transformation or semiparametric approaches in the TND framework.

In the absence of strong biological theory, it is unlikely that one will know the functional form of the protection function. The simulations using simple relationships show that if that relationship is misspecified in a parametric model, the result can depart strongly from the true relationship. For this reason, GAMs or similar semiparametric specifications are likely to be preferable in most instances for their robustness to the shape of the relationship. However, this flexibility also introduces trade-offs. GAMs are more susceptible to overfitting when the data are limited, and they require sufficiently large datasets with good coverage across the COP range, since they do not extrapolate reliably beyond the observed values. This observation aligns with Middleton and Larremore's finding that scaled logit performance declines sharply at small sample sizes; our simulations extend this characterization by evaluating performance across 125 to 5000 cases and similarly show improved recovery of the protection function as sample size increases. Some of the observed issues with uneven distribution of antibody titers may be artifacts introduced by the simulation's design of immunity waning and boosting mechanisms, although not all such issues are likely to be artificial. Future work could consider alternatives that incorporate penalization or priors.

Moreover, this study illustrates how using continuous immunity measures can bypass a known bias in TND-based vaccine effectiveness estimates for leaky vaccines. Because such vaccines offer partial protection to all recipients, natural immunity accumulates more rapidly among the unvaccinated.^13^ This process narrows the infection-rate difference between groups and causes the OR to drift toward the null. This pattern represents a specific manifestation of depletion of susceptibles. In contrast, modeling infection risk directly as a function of COP level does not rely solely on vaccination status and therefore sidesteps this biases, capturing how immunity itself influences susceptibility. Our simulations also incorporated the selection bias intrinsic to the TND, which arises from conditioning on symptomatic test-seeking individuals. Despite this sampling restriction, correctly specified transformed logistic models and adequately powered GAMs recovered the true COP–IRR relationship, suggesting that this form of selection did not materially distort estimation under the modeled scenarios. Overall, a TND can recover the COP–IRR relationship when either the correct functional form is used or a flexible semiparametric alternative is applied with sufficient data, in both homogeneous and heterogeneous risk settings. However, unlike analyses of vaccine effectiveness, causal interventions on the correlate may not be well defined, as no biologic mechanism exists to fix COP levels uniformly across individuals.

A strength of this study was the use of multiple protection functions, which allowed the identification of biases arising from model misspecification. The inclusion of a scenario with measured confounding under realistic simulation conditions is another strength. Several limitations should also be noted. First, the use of simple IRR and risk functions may have affected the granularity and generalizability of findings. We assume that the COP fully encapsulates protection, and that its relationship is the same whether COP levels are induced by vaccination, infection, or both. This assumption may be violated if similar COP levels reflect different underlying immune states, such as differences in antibody functionality or accompanying cellular immunity, across these pathways. Stratifying estimates by those with prior vaccination or not could partially mitigate this issue. Relatedly, the model simplifies assumptions about immunity boosting and waning, which may differ from patterns observed in real populations. Prior studies suggest that immunity waning could be nonlinear,^35^^,^^36^ although some of the effects are partially represented by the nonlinear protection functions. Second, antibody levels measured at the time of sampling were treated as exposure-proximal. If antibody levels begin to rise shortly after exposure but before sampling, and this rise occurs uniformly across individuals, the estimated protection function would be expected to shift along the COP axis while largely preserving its shape, while greater heterogeneity in post-exposure dynamics could instead induce bias. Additionally, the symptomatic fraction was assumed to be constant across COP levels. Variation in this fraction could shift the estimand from infection risk to symptomatic infection risk and alter the estimated protection. Third, the vaccination schedule was simulated as uniform over time. A more right-skewed rollout could improve realism but is unlikely to change the overall conclusions. Furthermore, the assumption of homogeneity among agents, including identical transmission characteristics and mixing patterns, may not reflect real populations.^38^ Finally, the approach requires TNDs with antibody measurements for both cases and controls, which are more feasible in prospective or specifically designed studies than in retrospective electronic health record settings.

Taken together, these findings highlight the central role of antibody levels as COP for evaluating infection risk across different study designs. When the protection function is correctly specified, logistic regression models incorporating natural logarithmic transformations of the IRR function accurately recover both linear and nonlinear relationships. When the underlying functional form is unknown, semiparametric approaches such as GAMs offer a flexible alternative, provided that sample sizes are sufficient and the COP values span an adequate range. These considerations are essential for designing TND studies that aim to characterize the relationship between exposure-proximal immunity and infection risk and for interpreting results in applications involving vaccine evaluation and other public health investigations.

## Supplementary Material

Web_Material_kwag036

## Data Availability

Code is available at: https://github.com/Ziyuan-Zhang429/TND-COP-IRR.
