# Supplementary material for: Use of the test-negative design to estimate the protective effect of a scalar immune measure: a simulation analysis
Source: Am J Epidemiol. 2026 Mar 4;195(5):1388–96. doi: 10.1093/aje/kwag036 (PMC13149011; doi:10.1093/aje/kwag036)
Supplement: Web_Material_kwag036 [file web_material_kwag036.zip › supplementary_data.docx]

## **Supplementary Material: Use of the test-negative design to estimate the protective effect of a scalar immune measure: A simulation analysis**

**Authors** Ziyuan Zhang, Christopher B. Boyer, Marc Lipsitch

**This file contains:**

**Figures S1-S3, Table S1**

### ****

### **Figure S1. Directed acyclic graph for Scenario 2**

**Figure S2. Sensitivity Analysis – Performance of exposure-proximal TND regression estimators using data from compiled simulation days 500 to 600 with an increment of 10**

**Panel A** used data from simulation where infection risk correlates with squared COP level, and **panel B** used data from simulation where infection risk correlates with cubic COP level.

### **Figure S3. Performance of exposure-proximal TND regression estimators across risk groups using data from a single simulated collection day versus aggregated collection days**

**Panel A** used data collected from simulation day 600 among those with normal risk and high risk, respectively; **panel B** used data from simulation days 500-590 with an increment of 10 among those with normal risk and high risk, respectively.

### **Table S1. Averaged mean absolute error^a^ of models trained on aggregated or single-day data across early and late pandemic phases with varying sample sizes, accounting for antibody level and risk status**

**Low-risk group**

| **Sample Day** | **Early Aggregated Days (Day 50-140)** | | | **Early Single Day (Day 150)** | | **Late Aggregated Days (Day 500-590)** | | | **Late Single Day (Day 600)** | |
| --- | --- | --- | --- | --- | --- | --- | --- | --- | --- | --- |
| **Number of Cases^b^** | 300 | 1250 | 5000 | 125 | 500 | 300 | 1250 | 5000 | 125 | 500 |
| Untransformed Logit | 0.1086 | 0.1084 | 0.1082 | 0.0959 | 0.0901 | 0.1225 | 0.1217 | 0.1218 | 0.1252 | 0.1238 |
| Transformed Logit | 0.0314 | 0.0291 | 0.0290 | 0.0356 | 0.0202 | 0.0194 | 0.0102 | 0.0060 | 0.0342 | 0.0197 |
| Untransformed GAM | 0.1005 | 0.1084 | 0.1106 | 0.1306 | 0.1191 | 0.0597 | 0.0336 | 0.0216 | 0.0908 | 0.0549 |
| Transformed GAM | 0.0425 | 0.0505 | 0.0630 | 0.0533 | 0.0434 | 0.0265 | 0.0132 | 0.0079 | 0.0730 | 0.0704 |

**High-risk group**

| **Sample Day** | **Early Aggregated Days (Day 50-140)** | | | **Early Single Day (Day 150)** | | **Late Aggregated Days (Day 500-590)** | | | **Late Single Day (Day 600)** | |
| --- | --- | --- | --- | --- | --- | --- | --- | --- | --- | --- |
| **Number of Cases^b^** | 300 | 1250 | 5000 | 125 | 500 | 300 | 1250 | 5000 | 125 | 500 |
| Untransformed Logit | 0.2105 | 0.1964 | 0.1923 | 0.2418 | 0.1796 | 0.2324 | 0.2136 | 0.2073 | 0.2645 | 0.2223 |
| Transformed Logit | 0.1328 | 0.0746 | 0.0523 | 0.2075 | 0.1072 | 0.1279 | 0.0641 | 0.0321 | 0.2035 | 0.1006 |
| Untransformed GAM | 0.1953 | 0.1833 | 0.1833 | 0.3385 | 0.2482 | 0.1686 | 0.0889 | 0.0535 | 0.2554 | 0.1421 |
| Transformed GAM | 0.1412 | 0.0941 | 0.0935 | 0.2249 | 0.1309 | 0.1349 | 0.0659 | 0.0336 | 0.2577 | 0.1803 |

^a^ 1,000 simulations for each model.

^b^ The number of infection cases sampled for model training included a mix of individuals at both normal and high risk.
